# Financial Burden in Adults With Chronic Illness in Switzerland: A Secondary Analysis of Qualitative Interviews Using Natural Language Processing and Topic Modeling

**DOI:** 10.2196/79290

**Published:** 2026-03-18

**Authors:** Giovanni Spitale, Julia Seinsche, Rosa M S Visscher, Andrea Schöpf-Lazzarino, Josip Jurisic, Federico Germani, Elena Alder, Nikola Biller Andorno, Karin Ribi, Bettina Schwind

**Affiliations:** 1 Institute of Biomedical Ethics and History of Medicine, University of Zurich, Canton of Zurich, Zürich, Switzerland; 2 Careum School of Health, Kalaidos University of Applied Sciences, Gloriastrasse 18a, Zürich, Canton of Zurich, 8006, Switzerland, 41 43 222 63 31; 3 Department for Public Health and Health Services Research, Institute for Medical Information Processing, Biometry, and Epidemiology, Ludwig-Maximilians-University Munich, Munich, Bavaria, Germany; 4 Pettenkofer School of Public Health, Munich, Bavaria, Germany; 5 Center for Health Literacy, Careum Foundation, Zürich, Canton of Zürich, Switzerland

**Keywords:** financial burden, chronic illness, natural language processing, topic modeling, qualitative data

## Abstract

**Background:**

Chronic illness may cause a financial burden that affects patients, their caregivers, and families. While international research, mostly from the United States, has largely focused on cancer-related financial hardship, less is known about whether financial distress due to other chronic illnesses exists, specifically in countries that have universal health insurance coverage, such as Switzerland.

**Objective:**

This study aims to provide insights into how financial burden is discussed by individuals living with chronic illness in Switzerland.

**Methods:**

Based on a natural language processing (NLP) approach, alongside topic modeling, a secondary analysis of 180 qualitative interviews of individuals living with chronic illness (dementia, chronic pain, multiple sclerosis, Parkinson disease, and rare diseases) from the Swiss Database of Individual Patient Experiences was conducted.

**Results:**

Key categories identified were money issues, disability insurance, general insurance concerns, and work and loss of income. Individuals living with dementia and Parkinson disease appear to be more concerned with money issues, whereas people living with chronic pain, multiple sclerosis, and rare diseases are more burdened by insurance-related concerns, specifically disability insurance-related challenges. Bureaucratic hurdles and employment instability appear to contribute to the financial burden of people living with chronic illness in Switzerland.

**Conclusions:**

Financial burden is a complex issue among individuals living with chronic illness in Switzerland. Our findings indicate that effectively addressing this burden requires a comprehensive and context-sensitive strategy. Targeted interventions should consider factors such as insurance eligibility, employment flexibility, and the mitigation of out-of-pocket costs to improve financial stability and quality of life for affected individuals.

## Introduction

Due to an aging population and improvements in the early detection and treatment of certain diseases, the number of people with chronic illnesses is expected to increase further in Switzerland [1], where chronic illnesses currently account for approximately 80% of direct health care costs [2,3]. These demographic trends are expected to be further amplified by ongoing technological advances, leading to a steady rise in health care costs for chronic illnesses.

Over the past decade, research on the financial aspects of chronic illness has gained momentum, particularly in the United States, where the lack of universal health insurance has led to health care debts becoming a serious challenge to families’ health and economic stability [4]. For example, 45%‐49% of people living with cancer in the United States report financial difficulties [5-7]. In other countries, cancer-related prevalence of financial burden ranges from 16% to 73% [7,8], probably depending on the type of health care system, as in countries with a publicly funded health care system, only 22%‐27% of study participants reported being affected by financial difficulties [8].

Much less is known about the financial burden among people living with other chronic illnesses. However, existing evidence indicates that approximately 70% of participants living with multiple sclerosis reported experiencing financial difficulties [9], compared to 45% of participants living with heart failure [10]. Overall, emerging evidence on financial burden appears largely to focus on specific chronic illnesses. This makes it difficult to understand whether and to what extent people with certain chronic illnesses experience financial burden differently from others, and how this relates to the welfare system. Such insights are crucial for improving our understanding of issues related to health equity, social cohesion, and the protection of groups in vulnerable situations.

Although the financial burden of chronic diseases has received considerable international attention over the past decade, to our knowledge, Switzerland has not yet fully attempted to understand or quantify it. Some studies have emerged that address financial toxicity in Swiss patients with cancer, for instance, identifying heightened financial burden among those with low incomes who live far from health care centers [11]. However, these studies have been limited in scope so far and continue to focus predominantly on cancer, which warrants a broader and more in-depth understanding of financial burden across chronic illnesses. This is even more the case given that, although Switzerland is a rich country on a global scale, wealth is unevenly distributed, and financial matters are considered taboo in Swiss society [12]. Establishing parallels for the Swiss context on the basis of international data is particularly challenging, given that the Swiss system is distinctive [13]: it is universal in coverage yet administered by private insurers, within a decentralized regulatory framework shaped by the influences of direct democracy [14]. This complex system carries the risk that individuals may not receive the financial support they need.

Therefore, the objective of this study is to obtain an initial understanding of whether financial burden exists across chronic illnesses in Switzerland and to identify patterns within and comparisons between chronic illnesses. The insights will contribute, in addition to other findings, to the development of a conceptual framework specific to Switzerland for assessing the extent of financial burden across different chronic illnesses thereafter. Further details on this framework’s development have been published previously [15].

## Methods

### Overview

**Figure 1.**
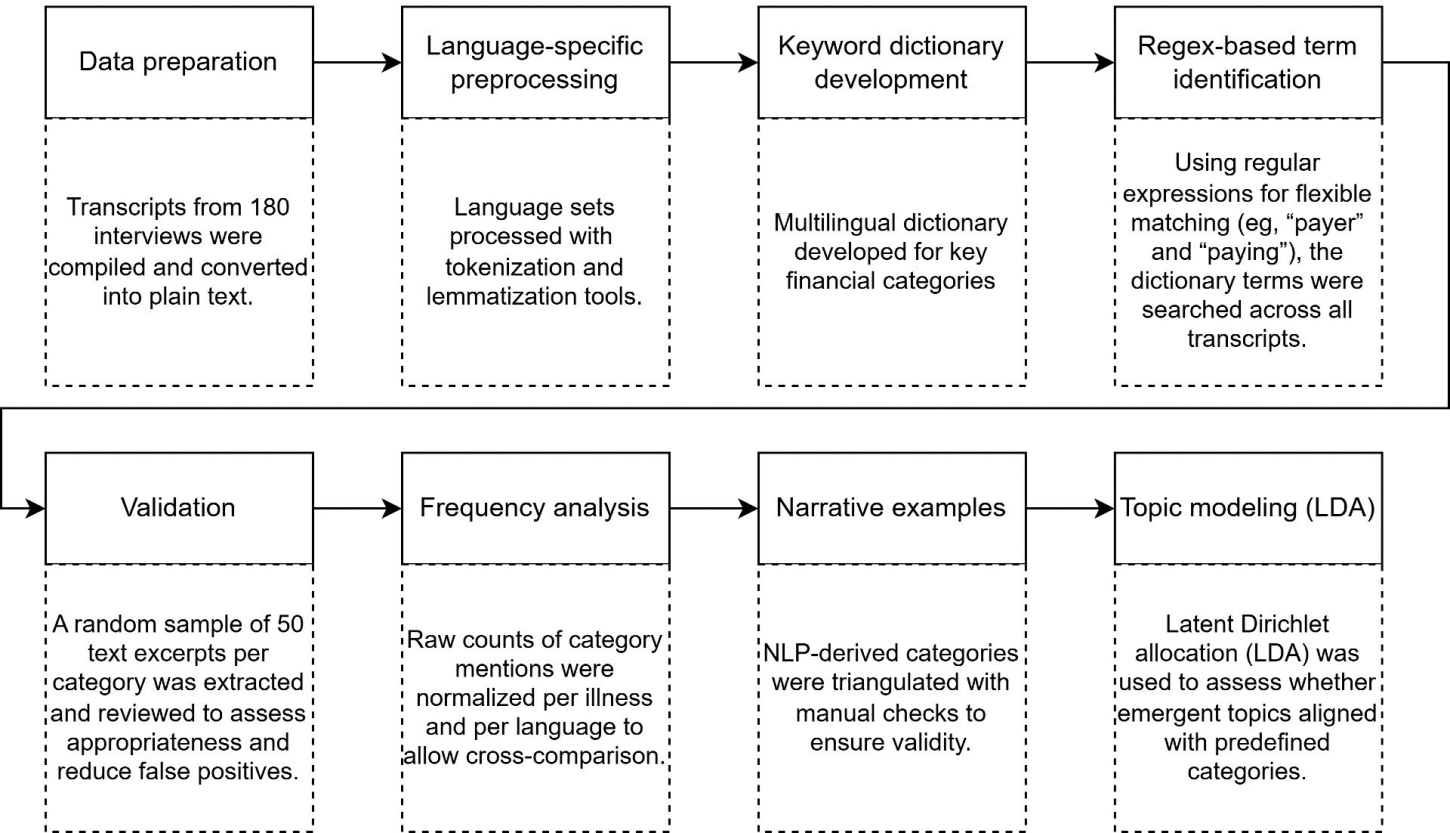
Natural language processing (NLP) pipeline for detecting themes of financial burden. This figure outlines the NLP workflow used to extract and analyze expressions of financial burden across the corpus. LDA: latent Dirichlet allocation; Regex: regular expressions.

Natural language processing (NLP) was applied to a large existing textual corpus of qualitative interviews with people living with various chronic illnesses in Switzerland. This provided a valuable opportunity to rapidly explore the relevance of financial burden on a large scale and in a novel way. The approach allows for the identification of patterns of financial burden while also providing illustrative narrative examples [16-18]. The NLP analysis was complemented by topic modeling, which uses the co-occurrence of words to discover latent topics [19]. The Python programming language (developed by Guido van Rossum) was applied to prepare, process, and analyze the textual data. A Zenodo repository (developed and hosted by the European Organization for Nuclear Research) is openly accessible and contains both the computational procedures (ie, the code base) and the anonymized passages to ensure full transparency and reproducibility [20]. The NLP pipeline of this study, which details the steps from text processing to analysis, is summarized in Figure 1. Reporting herein is guided by the SRQR (Standards for Reporting Qualitative Research) checklist [21].

### Dataset

The textual dataset used herein is based on qualitative interviews drawn from the Swiss Database of Individual Patient Experiences (DIPEx) Switzerland [22]. DIPEx Switzerland is committed to the systematic standards of DIPEx International to ensure the reliability and validity of country- and health condition–specific qualitative data [23].

We only included interviews with people who had experienced chronic illnesses in the dataset, while other conditions represented in the broader DIPEx dataset, such as experiences of pregnancy, were not considered. The included textual dataset consisted of 180 qualitative interviews with people living with dementia (n=32), chronic pain (n=34), multiple sclerosis (n=37), Parkinson disease (n=43), and rare diseases (n=34). Interviews were conducted either in 1 of the three main Swiss languages (German, French, Italian) or in English. They were gathered between 2018 and 2023 by researchers of different genders and professional backgrounds, who were either trained according to the international DIPEx standards or were senior health social scientists.

### Data Preparation

The textual dataset consists of the pseudoanonymized transcripts (.docx files), which were extracted using the textract library (PyPI, 2025b). Metadata (chronic illness, date of interview, language, and interview number) were retrieved from the file names. The extracted textual data were structured into a Pandas DataFrame [24], a spreadsheet-like data structure used in Python.

### Language-Specific Preprocessing

NLP was used with pretrained models from the open-source spaCy library for NLP in Python [25,26]. Models for German, French, Italian, and English were carried out separately to process the textual data according to the interview language. The de_core_news_md model was used for German, it_core_news_md and fr_core_news_md were used for Italian and French, and en_core_web_md was used for English. To reduce the number of word variations in the textual data, a lemmatization process was used. This process converts words into their most fundamental form, known as lemmas, to standardize the text and to support accurate data preparation for analysis [25]. Afterwards, text segmentation was used to divide the large texts into passages.

### Keyword Dictionary Development

In the next step, a comprehensive category and expression dictionary was developed to identify specific text passages related to financial burden. This dictionary was established through the generation of an initial list of German and English keywords selected by the research team. This list was subjected to a process of iterative translation (into French and Italian) and expansion through the review of interview transcripts. The process entailed the incorporation of linguistic variations, using regular expressions for flexible matching (eg, “payer” and “paying”), while allowing for the exclusion of keywords that identified text passages with no relevance to financial burden.

### Categories and Expressions

This list of categories and expressions with extracted text passages was converted into a DataFrame together with the respective metadata. The category and expression dictionary is as follows (refer to the codebase: [20]):

- General financial burden: captures expressions related to general financial difficulties and distress.
- Cost of care: includes expressions referring to the direct costs of medical care, treatments, and medications.
- Affording treatment: focuses on expressions related to the ability or inability to afford medical treatments.
- Insurance (general): addresses expressions related to health insurance coverage, premiums, deductibles, cost-sharing, and legal aspects.
- Insurance (disability): contains expressions related to disability insurance and pensions, including disability claims, compensation, and bureaucracy.
- Work and income loss: captures expressions reflecting loss of income due to illness, part-time and flexible work, job loss, and the ability or inability to continue work.
- Navigation: focuses on expressions associated with navigating the health care and social system, including case management and dealing with paperwork.
- Debt and loans: includes expressions related to financial debt and loans taken to cover medical or living expenses.
- Money issues: encompass expressions related to general financial concerns, such as having enough money, financial worries, and support.
- Other income: relates to alternative sources of income (pensions, savings, and salary).
- Social support: captures expressions related to social safety nets, including social security and social services.
- Anxiety: contains expressions that reflect financial anxiety, existential concerns, and fears about the future.

### Sampling and Validation

**Figure 2.**
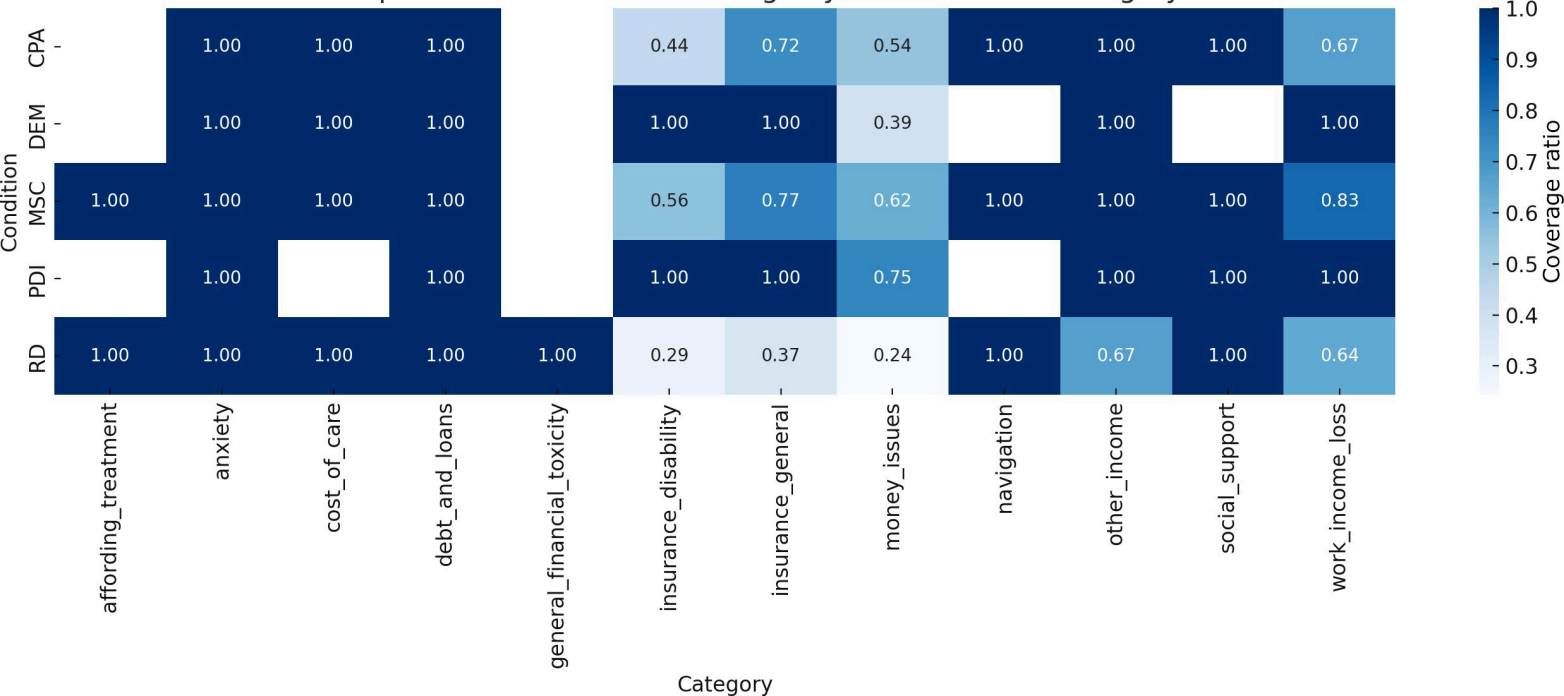
Heatmap of manual review coverage by condition and financial category. This heatmap illustrates the proportion of text passages manually reviewed for each combination of illness group (condition) and financial burden category. Darker shades indicate higher review coverage. Full review (100%) was applied to all low-frequency categories (≤50 passages), and a capped sample of 50 passages was used for higher-frequency categories. CPA: chronic pain; DEM: dementia; MSC: multiple sclerosis; PDI: Parkinson disease; RD: rare diseases.

To address the risk of selecting irrelevant text passages (false positives), we manually reviewed the extracted list of identified text passages and refined the dictionary, excluding terms that generated semantically inconsistent matches. All passages in categories with fewer than 50 extracted passages were reviewed in full. In categories with more than 50 extracted passages, 50 passages were selected at random and reviewed for relevance. Random selection of samples was conducted using Python’s random function, which chooses a specified number of unique elements from a sequence or set, without replacement. The relative coverage of the false positives check is reported in Figure 2; the data are available in this study’s repository [20].

Recognizing the risk of false negatives, we also conducted a manual review of 2 full transcripts per condition and language (33 interview transcripts in total), searching for semantically relevant expressions that had not been captured by the dictionary. These additional expressions were then integrated to improve sensitivity while maintaining thematic coherence.

To further strengthen contextual accuracy, for each identified match, the 3 preceding and 3 following text passages were extracted, providing a broader interpretive window during both manual validation and downstream analysis.

### Frequency Analysis

To quantify and compare the most frequently used categories and expressions within the extracted DataFrame, raw counts and normalized frequency (NF) analysis were used. NF analysis is widely used in text mining to detect the most frequently occurring expressions or categories [25] and to examine how these are distributed across text corpora, while accounting for text or document length.

#### Categories

First, the raw count of each category was calculated based on the extracted category and expression dictionary. To obtain normalized frequencies, the total raw count of each category was divided by the number of respective categories per chronic illness. This approach provides an overview of how the financial burden categories are distributed across the 5 chronic illnesses included in the study.

#### Expressions

First, the raw count of expressions was calculated based on the extracted category and expression dictionary. To produce normalized frequencies, the raw count of each expression was divided by the total number of expressions per category (considering language). This approach provides an overview of how the financial burden expressions are distributed across the different categories.

### Narrative Examples

To provide narrative examples, an NLP-based auto-coding approach was applied to the created category and expression dictionary [27]. For each category, we computed a thematic density score based on the number of matched expressions within each text passage contained therein. This score served as a proxy for thematic salience: passages containing multiple relevant expressions were considered denser. Within each category, passages were ranked by score, and illustrative examples were drawn from the top-scoring passages. Selection prioritized both semantic clarity and variation in chronic illness and experience. While this approach is not exhaustive, it aimed to balance narrative richness with analytic focus. A broader range of examples is provided in the Multimedia Appendix 1.

To protect participants’ anonymity, full interview transcripts are not publicly available but may be accessed in pseudo-anonymized form upon request.

### Topic Modeling

To examine whether the fine-grained terms align with broader topics across the full textual corpus, without the predefined categories, latent Dirichlet allocation (LDA) was applied. Topic modeling through LDA is commonly used for the analysis of health-related unstructured textual data [28,29]. It is an unsupervised machine learning technique that identifies clusters of words that occur together, referred to as “topics,” within a textual corpus [19]. It assigns each word to one or more latent topics based on the probability of this word’s co-occurrence with other words. This reveals their distribution patterns. Through iterative word-topic assignments, the algorithm optimizes the model to identify a stable distribution that best represents the latent structure of a textual corpus. The output includes a set of topics, each defined by a list of words. To use LDA for topic modeling, it is necessary to define the number of target topics in advance [19]. The number of topics was determined by following an exploratory phase in which models with fewer (3-4) and more (6-8) topics were tested and found either to collapse distinct themes or to produce semantically fragmented topics. Model quality was assessed qualitatively through topic interpretability and alignment with thematic structures in the interviews, and quantitatively through inspection of topic stability across multiple random initializations. Thereby, 5 topics per language were considered feasible to strike a balance between capturing a broad range of topics and ensuring the interpretability of the results. To preserve contextual richness and enable a full topic modeling process, the Gensim library’s LDA model was applied to the full corpus of interview transcripts included. While formal coherence scores were explored during model development, they were not used as a sole optimization criterion, as coherence metrics are known to correlate imperfectly with human interpretability in health-related qualitative corpora [30]. LDA was implemented using the Gensim library with standard priors (α and η set to “auto”), 1000 iterations, and multiple random seeds to assess robustness. Full details on hyperparameters, sensitivity analyses, and alternative model specifications, as well as interactive LDA visualizations, are provided in the accompanying Zenodo repository to ensure transparency and reproducibility [20]. The topics generated by the LDA model provided a computationally derived, yet thematically coherent, representation of how the financial issues were discussed in the interviews. Although abstracted, these topic clusters reflect tacit associations and co-occurrences that shape how chronically ill persons describe financial burden.

### Ethical Considerations

The Ethics Committee of the Canton of Zurich reviewed DIPEx Switzerland and determined that it did not fall under the Swiss law on research involving human participants (BASEC no 2018‐0050). Therefore, it was reviewed and approved by the internal review board of the Institute for Biomedical Ethics and History of Medicine at the University of Zurich (Switzerland, CEBES-2017‐09).

## Results

### Overview

A total of 1509 text passages were extracted across all chronic illnesses and languages: chronic pain (n=347), dementia (n=225), multiple sclerosis (n=304), Parkinson disease (n=138), and rare diseases (n=565). Most text passages were in German (n=1053), followed by French (n=288), Italian (n=127), and English (n=111), reflecting the number of DIPEx interviews per language, with about two-thirds coming from the German-speaking part of Switzerland. Table 1 provides a detailed breakdown of extracted passages by chronic illness and language. The results presented in the following focus on German-language data, because results across the Swiss language regions were similar. Details showing this similarity are provided in the Multimedia Appendix 1. We adopted this approach to make the results section more comprehensible and readable, while ensuring that no content was lost.

**Table 1.** Number of text passages extracted across languages and chronic illnesses.

Language	Chronic pain	Dementia	Multiple sclerosis	Parkinson disease	Rare diseases
German	242	171	190	90	360
French	72	46	74	15	81
Italian	33	8	40	11	35
English	—^a^	—^a^	—^a^	22	89

a Not applicable.

### Frequency Analysis

#### Categories

The most frequently mentioned category is “money issues*”* with an NF of 33.63%, followed by “insurance (disability)” (NF=19.81%). “General insurance concerns” (NF=16.92%) are followed by “work and income loss” (NF=15.38%). Less common categories are “other sources of income” (NF=7.51%) and “debts and loans” (NF=3.92%)*.*

**Figure 3.**
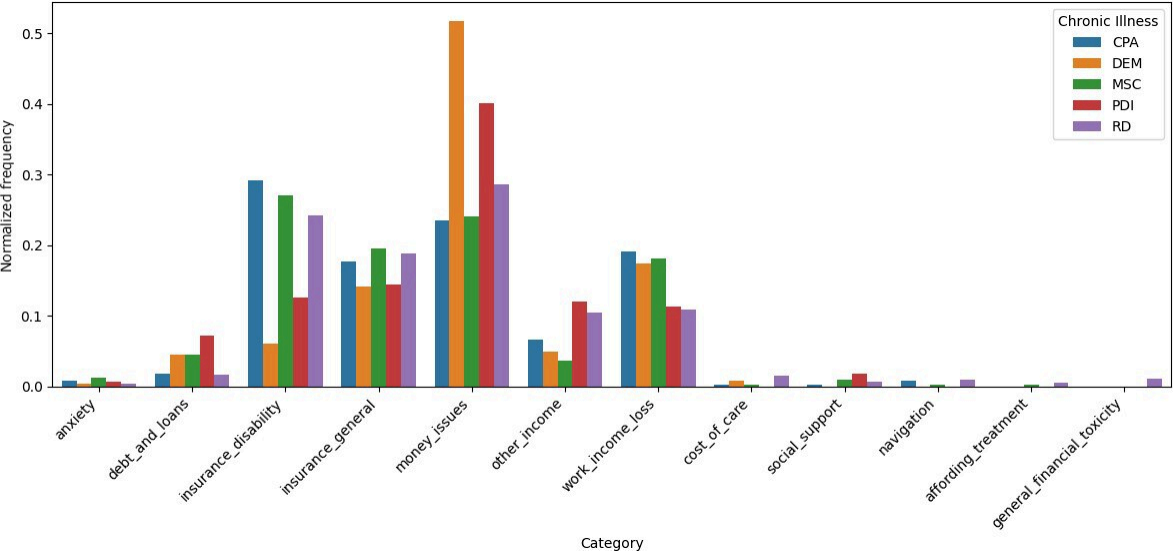
Histogram of normalized frequencies of categories per condition. This histogram visualizes the normalized frequency distribution (range 0‐1) of financial burden categories across 5 conditions: chronic pain (CPA), dementia (DEM), multiple sclerosis (MSC), Parkinson disease (PDI), and rare diseases (RD). Each bar represents the proportion of a category’s occurrence within the respective condition.

The normalized frequencies of categories demonstrate distinct patterns across the different chronic illnesses, pointing toward variations in the experience of financial burden within the context of the Swiss health and social system (refer to Figure 3): “money issues” are more frequently encountered by individuals living with dementia and Parkinson disease, while “insurance (disability)” and “loss of work and income” appear more frequently voiced by individuals living with chronic pain, multiple sclerosis, and rare diseases. In comparison, the categories of “cost of care” and “social support” appear to be mentioned less frequently. For the normalized frequencies of categories across chronic illnesses, refer to Table S1 in Multimedia Appendix 1. Deviations of category frequencies per chronic illness from the overall mean are provided in Figure S1 in Multimedia Appendix 1.

#### Expressions

Examining the most frequent expressions in each category provides an understanding of the vocabulary used to describe financial burden (Table 2). Expressions related to “insurance (disability),” “insurance (general),” and “money issues” were most frequently identified. The most prevalent German expression is invalidity insurance (IV; NF=18.61%), which is associated with the “insurance (disability)*”* category. This is followed by “Krankenkasse” (health insurance; NF=12.77%), which is associated with the “insurance (general)*”* category. Expressions such as “bezahlen” (to pay; NF=10.18%) and “kosten” (to cost; NF=8.01%) in the “money issues” category are most frequent, followed by the expression “Franken” (Swiss francs; NF=7.93%). Respective results for the other languages are provided in Table S2 and Figures S2-S5 in Multimedia Appendix 1. Taken together, the expressions suggest financial burden in chronic illness despite existing health and social insurance schemes.

**Table 2.** Overview of raw counts and the highest normalized frequencies of expression per category (German).

Category	Expressions	Raw count	Normalized frequency
Insurance (disability)	Invalidity insurance (IV)	223	0.1861
Insurance (general)	Krankenkasse (health insurance)	153	0.1277
Money issues	Bezahlen (pay)	122	0.1018
Money issues	Kosten (costs)	96	0.0801
Money issues	Franken (Swiss francs)	95	0.0793

### Narrative Examples

The top-scoring passages related to financial burden were predominantly found within the categories “money issues,” “insurance (disability),” and “work and income loss.” The following narrative example is intended to illustrate “money issues”:

*Ich sage die Materialien, die ich habe, sind natürlich nicht einfach wie ein einfaches Pflaster, wo man in der Migros kaufen kann. Es sind speziellere Sachen, die dementsprechend auch preislich höher. Und ja, ich sage, wenn ich jetzt viel hätte, wäre es für mich ein recht großer Kostenpunkt, wo ich nicht wüsste, wieviel kann ich von dem tragen. (I’d say the materials I use are, of course, not like a simple plaster you can buy at Migros (Swiss supermarket chain); they’re more specialized and, accordingly, more expensive. If I needed a lot of them, it would be a significant financial burden for me, and I wouldn’t know how much of it I could afford*).

[Row 1016, rare diseases 01]

This passage emphasizes that the practical challenges and costs of medical necessities go beyond shopping for groceries; it sometimes requires special supplies and health care items, the procurement of which is described as burdensome. From the analysis, it appeared that experiences of financial burden related to “insurance (disability)” may also be connected to administrative delays and complex bureaucracies:

*Ja, das war natürlich ziemlich schwierig, bis dann alles kam, auch von der SUVA [Schweizerische Unfallversicherungsanstalt]. Vor allem dieser ganze Papierkrieg… Also, es gibt einen Ordner voll mit SUVA und IV [Invalidenversicherung] und … ja. Das zog mich eben auch runter, mit diesen Finanzen. Denn die Rechnungen kamen ja trotzdem, man musste sie ja trotzdem irgendwie bezahlen. Und ich musste das alles von meinem Ersparten nehmen, bis heute. (Yes, it was quite difficult, especially waiting for everything to come through, even from SUVA [Swiss National Accident Insurance Fund]. Above all, the whole paper trail… I mean, there’s a whole binder full of stuff from SUVA and disability insurance. And yeah, that really dragged me down, financially. Because the bills kept coming; you still had to find a way to pay them. And I had to use all of my savings for that, and still do, to this day*.).

[Row 80, chronic pain 08]

This account reveals how delays in financial support due to administrative bureaucracy exacerbate the experienced distress, strain individual savings, and possibly contribute to experiences of poverty. This, in turn, can lead to feelings of devaluation, as exemplified below:

*Und das hört er, und dann schreibt er ins Protokoll hinein: “Sie freut sich,” also quasi, so schlecht kann es ihr nicht gehen. Das ist unglaublich. Ich habe das dann alles entsorgt nachher, ich wollte es nicht mehr lesen. Es war so, als ob er dachte: “Die macht jetzt wieder auf krank, die will einfach eine IV [Invalidenversicherung], weil sie das Gefühl hat ...” das fand ich schlimm. Und beim zweiten Vertrauensarzt, vom Taggeld aus, war es auch nicht besser. Der Neurologe hat zu mir gesagt: “In dieser Funktion können Sie sicher nicht mehr arbeiten. Wenn Sie so viel reden müssen, triggert das ja Ihre Beschwerden. Und anders kann man Ihnen auch nicht viel anbieten.” Und er sieht das schon, hat er gesagt. Im Protokoll stand dann das Gegenteil: “Sie kann im jetzigen Job sofort wieder anfangen.” (And he hears that, and then he writes in the report: “She’s happy,” meaning, basically, she can’t be doing that badly. It’s unbelievable. I threw it all away afterwards; I didn’t want to read it anymore. It was like, as if he thought: “She’s just pretending to be sick again, she just wants disability insurance because she thinks she deserves it…” That really upset me. And with the second insurance doctor, from the daily sickness benefit side, it wasn’t any better. The neurologist told me: “In this role, you definitely can’t work anymore. If you have to talk that much, it triggers your symptoms. And we can’t really offer you much else.” He clearly saw it. But in the report, it said the opposite: “She can return to her current job immediately.”*).

[Row 282, chronic pain 26]

This illustrated situation highlights how clinical recognition does not necessarily translate into recognition by the disability insurance system. Such contradictions may yet risk undermining trust, compounding burden, and perpetuating structural skepticism.

From the analysis, it appeared that work experiences and thus income loss (“work and income loss”) are also influenced by the Swiss insurance system:

*Ich hätte ja dann eine berufliche Eingliederung eigentlich, äh, zugute gehabt. Aber eben, man hat ja schon entschieden: Ich gehe in die Handelsschule, und die haben sie übernommen. Ich wurde nicht gefragt, ob ich das wirklich möchte oder ob das das Richtige ist — wie man das heute abklären würde. (I was actually entitled to vocational reintegration, but it had already been decided that I would go to business school, and they covered that. I wasn’t even asked whether I really wanted that or whether it was the right thing, like one would clarify today*.).

[Row 1357, rare diseases 18]

This example underscores how decisions regarding work and training are, at times, made unilaterally by insurance providers, without considering the affected individual’s agency or aspirations. Anticipating such negative experiences, others reported avoiding insurance support altogether:

*Nein, selber habe ich mir, ehrlich gesagt, die Erfahrung erspart, weil ich von anderen Personen weiß, wie schwierig das ist. Äh, es ist ein Kampf. […] Man bekommt halt einfach — je nachdem — wirklich fast nichts oder gar nichts an Unterstützung. […] Ich habe einfach Angst gehabt, dass ich nachher wie abgestempelt werde. (No, to be honest, I spared myself the experience, because I know from others how difficult it is. It’s a struggle. You end up getting almost nothing or nothing at all. […] I was just afraid that I’d be labeled—that they’d say I’m fully able to work, and then my employer would assume I have to work full-time because I’m now labeled as 100% able to work*.).

[Row 183, chronic pain 15]

This narrative example shows the fear of stigmatization and discrimination, as well as the fear of impairment and psychological suffering. Applying for a reduced earning capacity pension may not lead to a positive outcome; instead, a devaluation of one’s own experiences is expected, followed by increasing work pressure. The reduction in work and the associated loss of income are therefore borne by the individuals themselves and by the flexibility provided by their employers:

*[…] Ähm, ja, da bin ich eigentlich sehr dankbar. Da habe ich einen sehr flexiblen Arbeitgeber. Und ähm, ja, ich bin jetzt mittlerweile Ausbilder für Physiotherapeuten oder begleite Physiotherapeuten. Da habe ich natürlich auch Verständnis von den Kollegen. Das ist alles im medizinischen Bereich — die wissen, was mit mir los ist*. (Um, yes, I’m actually very grateful, I have a very flexible employer. And um, yes, I’m now a trainer for physiotherapists, or accompany physiotherapists. Of course, I still have understanding from my colleagues. It’s all in the medical field, they know what’s going on with me.).

[Row 5, chronic pain 01]

This case illustrates the importance of workplace flexibility and support in mitigating the financial impact of illness. Together, these narrative examples illuminate a landscape where formal support for work reintegration is often insufficient, inaccessible, or accompanied by fear of stigma and discrimination and invalidation of experiences.

### Topic Modeling

The LDA-based analysis identified 5 key topics, highlighting distinct concerns across chronic illnesses that substantially align with the reported frequencies of categories. These topics include references to cost, work, health care providers, and bureaucratic navigation—components already identified as prominent, while also revealing subtle nuances beyond the dictionary approach. More specifically, the five identified LDA topics comprise (1) financial concerns, (2) work and mobility, (3) role of health care providers, (4) health care expenses, and (5) interplay between insurance entitlement and work. Topic tables across the different language corpora and interactive LDA visualizations are available in the study repository [20], and a detailed analysis is available in Multimedia Appendix 1; here, we focus on the German subcorpus.

Topic 1*, “*financial concerns*,”* combines words such as “bezahlen” (to pay), “Krankenkasse” (health insurance), and “Geld” (money) with “irgendwie” (somehow) and “lachen” (laugh), indicating possible uncertainty and insecurity in expressing financial burden due to chronic illness in Switzerland. Topic 2, “work and mobility,*”* encompasses the terms “Auto” (car), “Arbeit” (work), and “Leute” (people). This thematic connection potentially underscores the significance of transportation in facilitating social and professional interactions. Topic 2 also comprises words such as “selber” (oneself) and “denken” (to think), which may indicate an emphasis on autonomy and self-sufficiency. Topic 3, “role of health care providers,” includes words such as “Arzt” (doctor), “brauchen” (to need), and “bekommen” (to receive). These words likely highlight the important role of health care providers and the needs and expectations of patients with chronic illnesses toward them. The term “bezahlen” (to pay) presumably emphasizes the financial aspects associated with this medical care. Topic 4 focuses on “health care expenses” and includes words such as “zahlen” (to pay), “Franken” (Swiss francs), “Krankenkasse” (health insurance), “IV,” and “bekommen” (to receive). These words suggest both financial burden and support through insurance. The topic also contains words such as “arbeiten” (to work), which may show the connection between these benefits and employment. Words such as “irgendwie” (somehow) and “Sache” (thing) appear to hint at uncertainties, and “lachen” (to laugh) may express irony, frustration, or sarcasm. Topic 5, “interplay between insurance entitlement and work*,”* includes words such as “arbeiten” (to work), “selber” (oneself), “IV,” “bezahlen” (to pay), and “Krankenkasse” (health insurance), which, in combination with “denken” (to think) and “irgendwie” (somehow), may indicate the independence required to find ways to manage work and financial aspects on one’s own despite chronic illness.

## Discussion

### Principal Findings

This study provides an initial insight into perceptions of financial burden across chronic illnesses in Switzerland, warranting further discussion. The NLP analysis of an existing large corpus of qualitative interviews revealed that the 4 most frequently mentioned categories were “money issues,” “insurance (disability),” “insurance (general),” and “work and income loss.*”* These are confirmed by the topic modeling and the top-scoring passages and the respective narrative examples, which are centered around the same categories, in particular, disability insurance.

### Money Issues

The category “money issues*”* stood out across all chronic illnesses. This finding aligns with previous studies that have emphasized the financial burden placed on people with chronic illnesses and their families internationally [31-33] and in Switzerland [14,34]. The participants’ narratives equally highlight the struggle with finances, a finding that should be taken seriously given the negative effects of financial strain on mental health as well as on physical and emotional well-being [10,35-39].

“Money issues” were frequently mentioned by people with dementia, Parkinson disease, and rare diseases. Regarding the former 2, 1 explanation might be that both illnesses primarily affect older adults [40,41] and are associated with a higher need for care. In Switzerland, previous studies found that 57% of people with dementia require daily or even around-the-clock care [42]. Concordantly, costs related to institutional care as well as indirect informal care costs, such as opportunity costs and health expenses, have been identified as the main cost drivers in dementia-related expenses in Switzerland and abroad [42-45]. While, to the best of our knowledge, detailed information on the informal care of people with Parkinson disease in Switzerland and related costs is lacking, findings from international studies suggest a similar pattern. In the United Kingdom, for example, formal domestic and personal care were accessed by only 25% each, while 80% relied on informal carers [46], resulting in substantial direct and indirect costs for both people with Parkinson disease and their caregivers [47,48]. Overall, the need for costly formal and informal care in people with dementia and Parkinson disease may explain the prominence of “money issues*”* in both illnesses.

People with rare diseases face distinct and multifaceted problems related to “money issues.” Obtaining a correct diagnosis is typically a long and costly process, involving numerous medical appointments. In Switzerland, 41% of patients initially receive a false diagnosis, and 25% are correctly diagnosed only after 5 years, with diagnostic delays of up to 30 years reported in some cases [49,50]. Earlier diagnosis would not only reduce patient suffering but also lower resource usage and health care costs [51]. Even after diagnosis, treatment options remain limited. Only about 10% of people with rare diseases can be treated. Approximately 90% of these patients rely on off-label therapies [50], which are only partially reimbursed by health insurers. The same applies to many orphan drugs, that is, expensive drugs developed specifically for certain rare diseases, for which legal uncertainties persist in Switzerland regarding reimbursement and the criteria used to determine coverage [49-52].

### Disability Insurance

*“*Disability insurance*”* is another frequently mentioned category, which is strongly emphasized in the modeled topics and narrative examples, most of which deal with disability insurance. In Switzerland, the guiding principle of the disability insurance system is “rehabilitation before pension” [53], meaning that disability pensions are only granted if rehabilitation and work integration options aiming to restore working capacity have been exhausted [53,54]. Previous research found that people applying for disability insurance in Switzerland perceived an unequal distribution of power, as disability insurance agents are authorized to end financial support if rehabilitation measures or insurance requirements are not met. Meanwhile, applicants saw only limited opportunities to negotiate their own rehabilitation and occupational path [55]. Other major criticisms included the opacity of placements on the disability scale and the inappropriateness of training courses or rehabilitation measures [55]. These issues are reflected in the narrative examples and topic 4 of our analysis, in which frustrations with disability insurance processes were expressed, together with the resulting emotional toll. However, it must also be considered that returning to employment through disability insurance measures allows for individuals’ social reintegration, improving their quality of life [55,56].

Disability insurance was mentioned particularly often in relation to chronic pain, multiple sclerosis, and rare diseases. All 3 conditions often have an early onset [50,57-59]. Consequently, those affected frequently have not contributed sufficiently to the social security system to qualify for an old-age pension (state pension) and may also not have worked for a long enough period to build up substantial occupational pension or private benefits. These factors make them highly dependent on disability insurance.

The assumption that age is an important factor related to financial burden is supported by the fact that disability insurance was mentioned less often in the context of dementia and Parkinson disease. These conditions typically occur in older adults who have spent more years in the labor market and are therefore better covered by the 3 types of pension. However, once individuals reach retirement age, they are no longer eligible for disability insurance. This might be disadvantageous, as older adults in need of long-term care must finance most of these costs by themselves, with health insurance and the state pension covering only about 35% of the long-term care expenditures. In contrast, disability insurance and the government cover about 79% [14] for people with disabilities who have not yet reached retirement age and are therefore eligible for disability insurance. Therefore, for older people with dementia or Parkinson disease, the transition from disability insurance to an old-age pension can indeed result in a financial disadvantage.

In the case of multiple sclerosis, the progressive nature of the illness is another important factor, requiring expensive treatments and resulting in high rates of disability [9,60]. Findings from Switzerland indicate that the costs of multiple sclerosis are closely related to the severity of the disease and increase steadily with it [61,62], mainly driven by drug expenditures in early disease stages [61-63]. Furthermore, multiple sclerosis ranks among the diseases with the greatest increase in health care spending in Switzerland over the past decade [2]. Similarly, in the United States, multiple sclerosis is associated with the highest total out-of-pocket expenditures compared to other chronic illnesses and can even lead to medical bankruptcy [64]. Taken together, these findings underscore the importance of support from disability insurance and other social welfare systems.

Regarding rare diseases, many manifest early in life and are classified in Switzerland as “Geburtsgebrechen” (congenital disorders). In such cases, financial responsibility initially lies with the disability insurance (IV), which covers the costs of medical measures related to recognized birth defects up to the age of 20 years. However, reimbursement by the IV is dependent on a specific list of recognized congenital disorders (“Liste der Geburtsgebrechen*”*) and a corresponding list of medicines. After the age of 20 years, responsibility typically shifts to mandatory health insurance. Navigating the administrative processes of this transition can be difficult and may lead to even more insecurity regarding reimbursement [50].

### General Insurance

The third frequently mentioned category, “insurance (general),” includes an array of issues and seems to be similarly important across all chronic illnesses. According to narrative examples, major systemic barriers related to insurance are eligibility issues, insurance denials for people with pre-existing chronic conditions, and health insurance coverage. Although health insurance companies (37.7%) and the government (21.2%) together cover more than half of total health expenditures in Switzerland, a significant proportion of health care is financed by households’ out-of-pocket payments (21.3%) [34], which are notably higher than in many other European countries [65]. For chronically ill people, it is particularly important that the total costs of copayments for outpatient treatment in Switzerland are capped [14], which is not the case for inpatient copayments. On the other hand, as Desborough et al [66] point out, people may also struggle to reach the copayment threshold. The narrative examples and topics 1 and 4 illustrate that these insurance-related barriers can lead to psychological distress—an issue confirmed in international literature [66-69].

### Work and Income Loss

The category of “work and income loss*”* reflects the impact of chronic illness on the ability to maintain employment and income. It highlights the indirect financial consequences of chronic illnesses, which are often as critical as direct medical costs [47,70]. Loss of employment can further exacerbate existing financial burden [66], forcing chronically ill people and families into precarious living situations [71,72]. Another work-related issue that was underscored in the narrative examples relates to work flexibility. Concerns were expressed about unrealistic workplace expectations. This is equally emphasized in the literature [73], stressing how understanding in the workplace can alleviate some of the burdens associated with chronic illness.

The normalized relative frequency of this category was highest in people with chronic pain, dementia, and multiple sclerosis. Regarding chronic pain and multiple sclerosis, a possible explanation is again that many young or middle-aged adults are affected, who are still working, making “work and income loss*”* a relevant concern for them. Indeed, multiple studies from Switzerland and other countries have reported a decline in income in people with chronic pain [74-78] and multiple sclerosis [61] due to factors such as reduced working hours, job changes, absenteeism including sick leaves, resulting job loss, or early retirement. Particularly in more advanced stages of multiple sclerosis, absence from work, reduced productivity, and loss of employment have been identified as major drivers of illness-related costs [61,62]. These findings again underscore the importance of disability insurance to cover these costs or support work reintegration.

The relatively high frequency of words related to “work and income loss*”* in dementia may be explained by the role of informal caregivers in dementia care. Caregivers are also affected by work and income loss and face high costs, such as opportunity costs and health expenses [42,45]. In Switzerland, it has been shown that indirect costs, or opportunity costs through informal caregiving, respectively, account for approximately 44% of the total cost of dementia [42].

### Topic Modeling

Overall, the topic modeling confirmed findings from the normalized frequencies analyses and narrative examples, demonstrating recurring concerns about health care costs, insurance coverage, and IV across multiple topics. The LDA results added value by surfacing cross-cutting patterns not captured by the predefined categories alone, for example, the entanglement of employment, autonomy, and insurance navigation that emerges as a latent theme across topics 2 and 5.

The frequent co-occurrence of work-related terms with bureaucratic and financial concerns reinforces the idea that these are interlinked experiences. Furthermore, the presence of emotionally charged and ambiguous terms (eg, somehow and laugh) in multiple topics suggests not only the material but also the affective complexity of financial burden and chronic illness. These findings align with prior research indicating that financial burden includes both economic hardship and its psychological correlates [36], while also validating and extending our frequency-based analysis through a different analytic lens.

### Limitations

The study is subject to certain limitations. A major limitation is that it relies on a corpus of semistructured interviews from the Swiss DIPEx database that were collected before the study. The interviews covered a variety of topics, some of which were more comparable to each other than others. However, all interviewees had one thing in common: they had all experienced illness within the Swiss health care system. On the other hand, although certain topics addressed in the interviews varied, they related specifically to the participants’ experiences of illness, which might have improved the validity [79]. The maximum diversity method was used for all DIPEx interviews included. This approach guarantees maximum variability in perspectives, but not representativeness [80]. Thus, the results provide valuable in-depth insights into the diversity of experiences but may not be generalizable. However, the findings are intended to inform the development of a conceptual framework that is based primarily on international literature and qualitative data from Switzerland, enabling broader generalization in subsequent research phases. Finally, no demographic variables from the participants were reported. Therefore, we could not examine potential differences between demographic groups. Prior research suggests that financial burden may vary between genders and across age groups [81], which is why the interpretability of our findings is limited. However, this information was not possible to retrieve from the DIPEx database. Nevertheless, this analysis was key to us in order to gain an initial impression of the different experiences in Switzerland, as the financial burden in relation to certain illnesses is still unknown [2].

### Conclusions

In conclusion, our findings show that the financial burden in chronically ill people is not a singular issue but rather a multifaceted challenge that intersects with bureaucratic barriers, high out-of-pocket expenses, employment insecurity, insurance-related challenges, and health care access. Given the exploratory nature of this study, these results should not be interpreted as calls for systemic reform. Instead, they highlight specific areas that may warrant closer attention in future research and stakeholder discussions. For instance, simplifying and clarifying disability insurance procedures may be particularly relevant for individuals with fluctuating or less visible conditions, such as chronic pain, multiple sclerosis, or rare diseases, whereas people living with dementia or Parkinson disease may benefit from clearer guidance on available financial supports. Employment instability also emerged as a contributor to financial strain, suggesting that improved communication between employers, insurers, and affected individuals could be a promising area for further exploration.

Overall, addressing issues related to financial burden in chronically ill people requires a comprehensive and context-sensitive approach. This study provides a first step toward such an approach and informs the next phases of our larger research project [15]. As described above, the results will be integrated with international evidence from a scoping review and new qualitative interview data to develop a conceptual framework on financial burden and chronic illness specific to Switzerland, refined through stakeholder discussions. This framework will then guide the creation of a survey to measure financial burden in chronically ill individuals in Switzerland. Ultimately, survey results will be discussed with stakeholders to identify actionable solutions and potential changes to current policies and practices.

Through this stepwise process, our work lays the foundation for evidence-based discussions on how the Swiss health and insurance systems might better support individuals with chronic illnesses without overstating the conclusions that can be drawn from this exploratory study.

## Supplementary material

10.2196/79290 Multimedia Appendix 1 Results across German, French, Italian, and English text corpora.

## Abbreviations

DIPEx: Database of Individual Patient Experiences
IV: invalidity insurance
LDA: latent Dirichlet allocation
NF: normalized frequency
NLP: natural language processing
SRQR: Standards for Reporting Qualitative Research
