# Supplementary material for: Financial Burden in Adults With Chronic Illness in Switzerland: A Secondary Analysis of Qualitative Interviews Using Natural Language Processing and Topic Modeling
Source: JMIR Form Res. 2026 Mar 18;10:e79290. doi: 10.2196/79290 (PMC12998611; doi:10.2196/79290)
Supplement: Multimedia Appendix 1 [file formative-v10-e79290-s001.docx]

**Financial Burden and Chronic Illness in Switzerland: Using Natural Language Processing and Topic Modeling on a Large Corpus of Qualitative Interview Data**

## **Categories by Chronic Illness**

**Table S1.** Normalized category frequencies across chronic illnesses.

| Categories | Chronic Illnesses | | | | |
| --- | --- | --- | --- | --- | --- |
|  | CPA^a^ | DEM^b^ | MSC^c^ | PDI^d^ | RD^e^ |
| Affording treatment | NaN | NaN | 0.003012 | 0 | 0.005587 |
| Anxiety | 0.007673 | 0.004049 | 0.012048 | 0.005988 | 0.004190 |
| Cost of care | 0.002558 | 0.008097 | 0.003012 | 0 | 0.015363 |
| Debt and loans | 0.017903 | 0.044534 | 0.045181 | 0.071856 | 0.016760 |
| General financial toxicity | 0 | 0 | 0 | 0 | 0.011173 |
| Insurance disability | 0.291560 | 0.060729 | 0.271084 | 0.125749 | 0.241620 |
| Insurance general | 0.176471 | 0.141700 | 0.195783 | 0.143713 | 0.188547 |
| Money issues | 0.235294 | 0.518219 | 0.240964 | 0.401198 | 0.286313 |
| Navigation | 0.007673 | 0 | 0.003012 | 0 | 0.009777 |
| Other income | 0.066496 | 0.048583 | 0.036145 | 0.119760 | 0.104749 |
| Social support | 0.002558 | 0 | 0.009036 | 0.017964 | 0.006983 |
| Work and income loss | 0.191816 | 0.174089 | 0.180723 | 0.113772 | 0.108939 |

Note. The values represent the proportion of each category's occurrence relative to the total number of categories within the respective chronic illness. Higher values indicate a greater prominence of a particular financial burden theme within that condition;

^a^Chronic Pain, ^b^Dementia, ^c^ Multiple Sclerosis, ^d^Parkinson’s Disease, ^e^Rare Diseases

**Normalized Frequency Analysis**

**Table S2.** Most frequent expressions by category and language (FR, IT, EN).

| Language | Categories | Expressions | Raw counts | Normalized frequency |
| --- | --- | --- | --- | --- |
| French | Money issues | payer | 54 | 0.151261 |
|  | Insurance - disability | handicap | 41 | 0.114846 |
|  | Insurance - general | assurance maladie | 32 | 0.089636 |
|  | Debt and loans | prêt | 30 | 0.084034 |
|  | Insurance - general | caisse maladie | 30 | 0.084034 |
| Italian | Money issues | pagare | 36 | 0.251748 |
|  | Insurance - general | cassa malato | 29 | 0.202797 |
|  | Insurance - disability | invalidità | 18 | 0.125874 |
|  | Other income | pensione | 13 | 0.090909 |
|  | Money issues | costo | 10 | 0.06993 |
| English | Money issues | pay | 54 | 0.348387 |
|  | Other income | pay | 54 | 0.348387 |
|  | Money issues | cost | 23 | 0.148387 |
|  | Insurance - disability | iv | 12 | 0.077419 |
|  | Money issues | franc | 12 | 0.077419 |


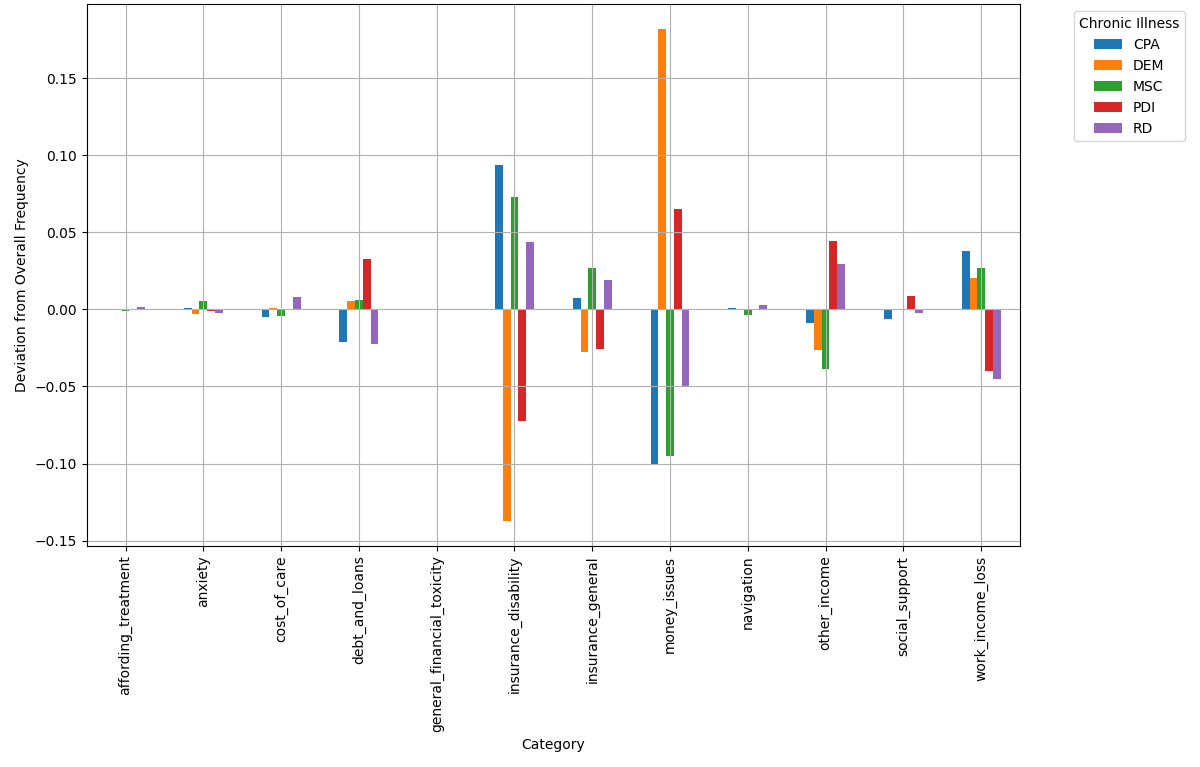
**Figure S1.** Deviation of category frequencies per chronic illness from the overall mean. This bar chart illustrates the extent to which the normalized frequency of categories within each chronic illness (CPA, DEM, MSC, PDI, RD) deviates from the overall mean normalized frequency across all conditions. Positive deviations indicate that the category is more frequently mentioned in a particular chronic illness compared to the average, while negative deviations suggest it is less prominent.

## **Expressions (FR, IT, and EN)**

In the **French** subcorpus, the most prominent expressions are related to *“handicap”* and “ invalidité” (disability) (combined frequency: 19.60%). Financial concerns also focus on *insurance* and *money issues*: the terms *“assurance” (insurance)* and *“caisse maladie” (health insurance)* also recur (combined frequency: 17.36%). Study participants also mention *“payer”* (to pay), 15.12%, mirroring the German results in their concern for everyday expenses, and *“prêt”* (loan), 8.40%.

The **Italian** interviews show a similar emphasis on *insurance,* with expressions like *“cassa malati” (health insurance)* and “*assicurazione*” (insurance) (combined frequency: 25.16%). *Money issues* are also prominent, represented by expressions such as *“pagare”* (to pay), 25.17%. but also bring attention to specific terms related to disability (*“invalidità”)*, 12.58%.

Although the **English** interviews are less frequent in the dataset, the same financial themes emerge. Words like *“pay”*, 34.83%, *“costs”, 14.83%,* and *“iv”* (invalidity insurance)*, 7.74%,* are among the most common expressions, confirming that financial difficulties due to illness is a shared experience across languages and chronic illnesses.

The following figures (Figures S2-S5) display the five most frequent expressions within each language, normalized by the total number of expressions in that language, and grouped by semantic category. This normalization approach ensures that intra-language frequencies are directly comparable across categories, independent of differences in corpus size or chronic illness distribution. Expressions were extracted from annotated text segments, aggregated per language and category, and normalized to yield proportions reflective of their salience within the language's overall discourse.


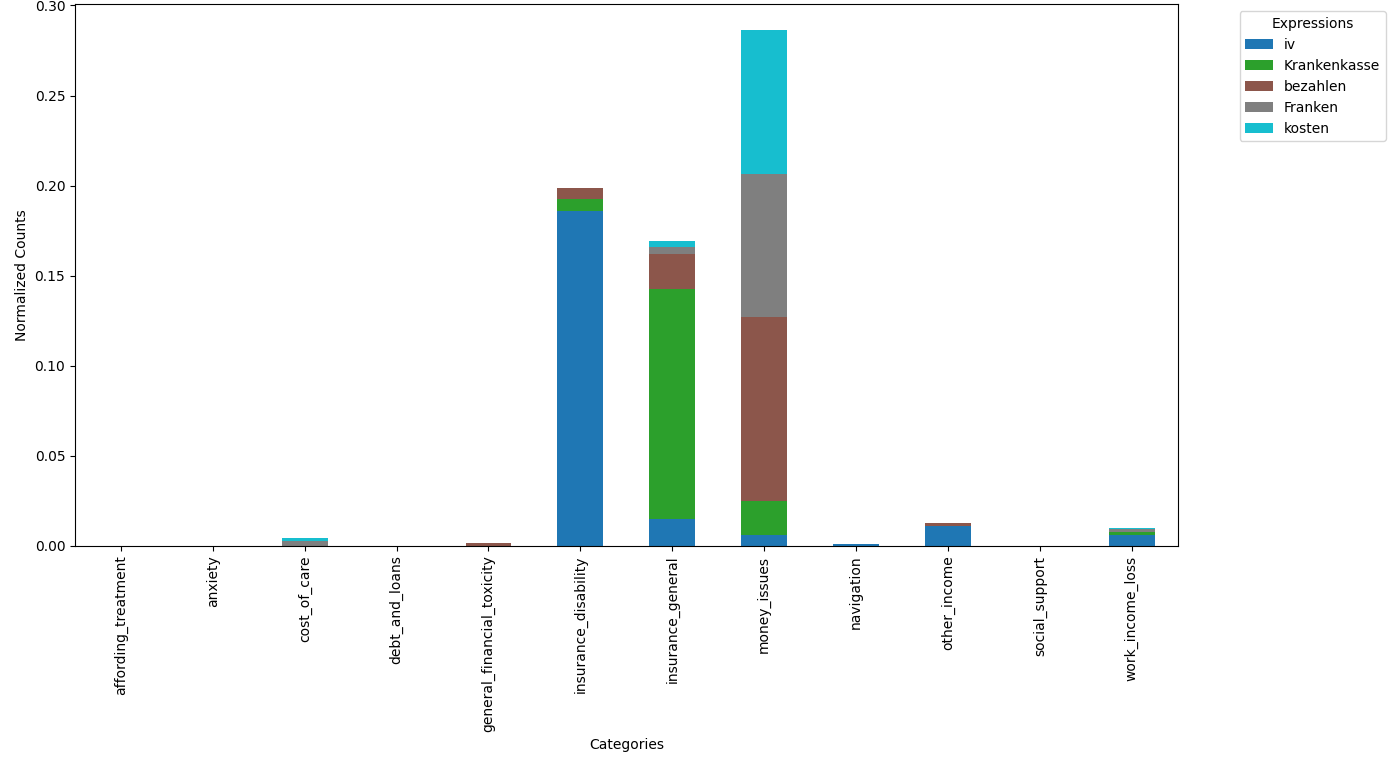
**Figure S2**. Top 5 normalized German expressions by category using language-level normalization.


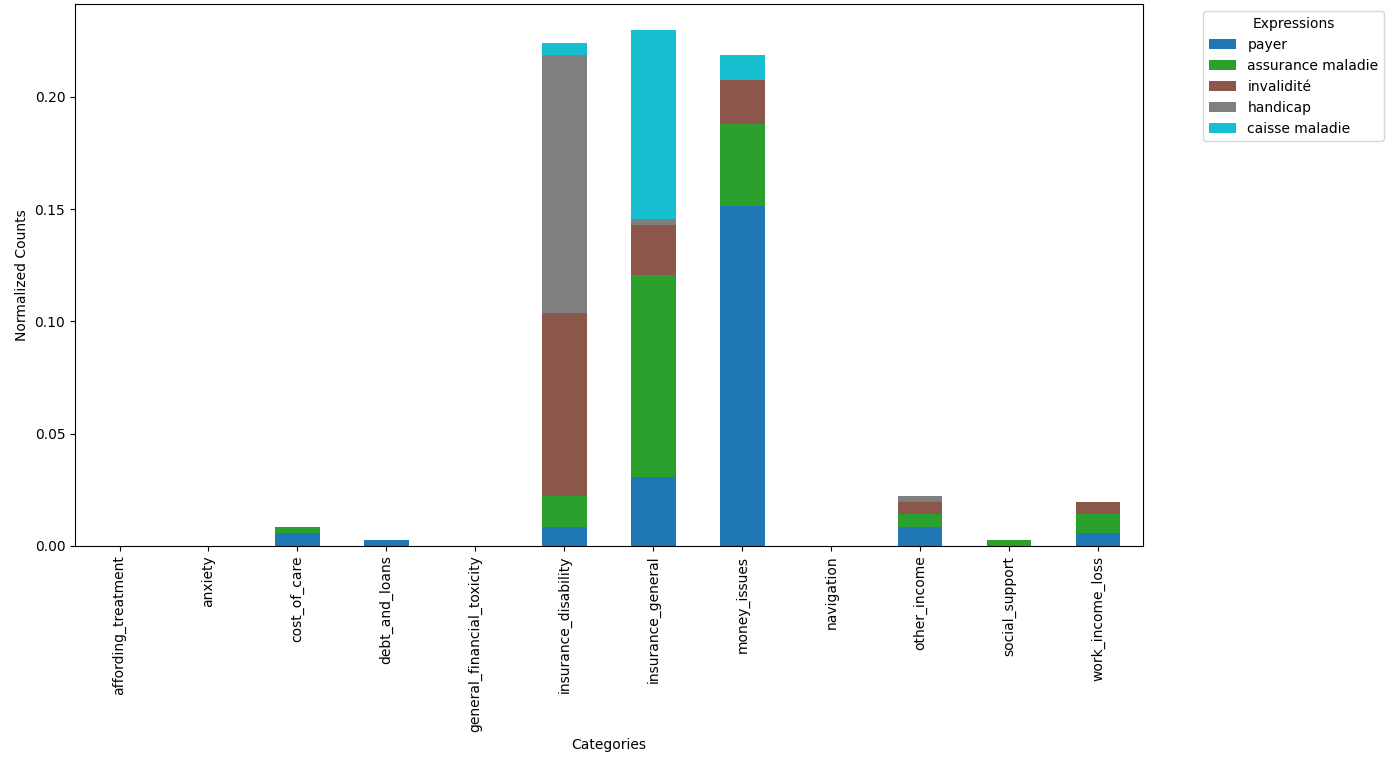
**Figure S3.** Top 5 normalized French expressions by category using language-level normalization.


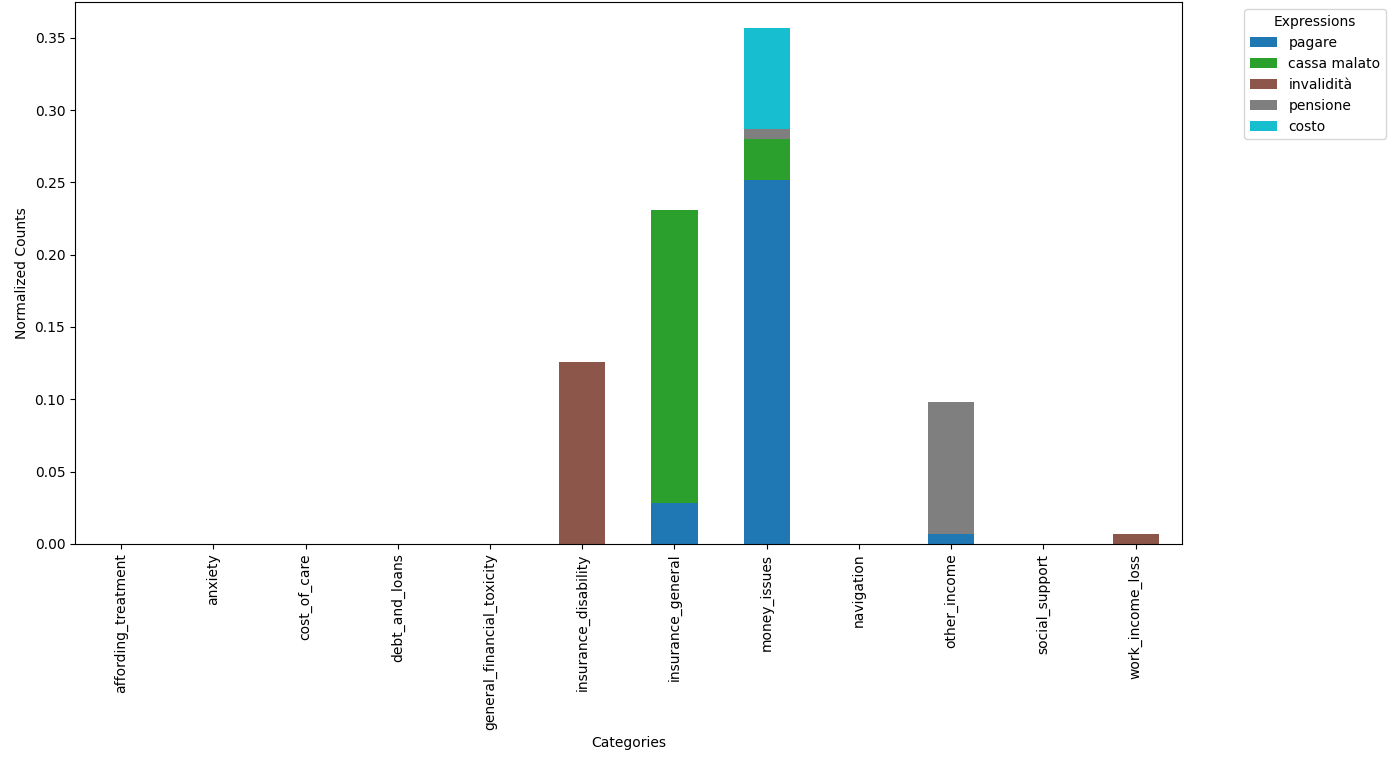
**Figure S4**. Top 5 normalized Italian expressions by category using language-level normalization.


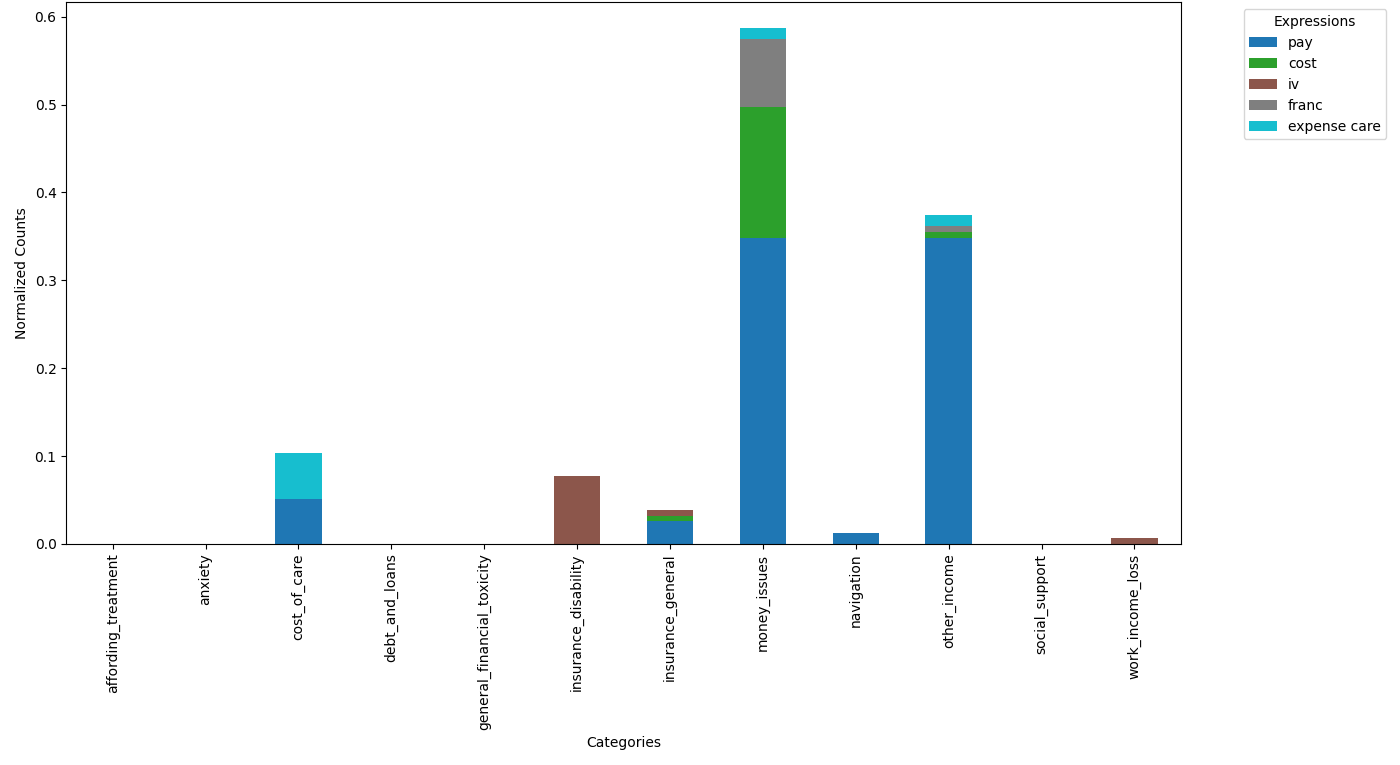


**Figure S5.** Top 5 normalized English expressions by category using language-level normalization.

## **LDA Topics (FR, IT, and EN)**

The **French** topic modeling output reveals themes primarily centered around healthcare, work, and financial challenges, reflecting common concerns in managing health and financial stability. **Topic 1** focuses on the role of healthcare professionals and assistance, with terms like "maladie" (illness), "médecin" (doctor), and "assurance" (insurance) suggesting a focus on study participants’ experiences with the healthcare system. Words like "aide" (help) and "trouver" (find) indicate seeking necessary support. **Topic 2** highlights the interplay between work and life, with words like "travailler", “travail” (work), and "vie" (life), reflecting the impact on work and daily life. The inclusion of "incompréhensible" (incomprehensible) and "niveau" (level) may suggest frustrations or difficulties in understanding or navigating work-related issues. **Topic 3** centers around medical care and insurance, with "médecin" (doctor), "maladie" (illness), and "assurance" (insurance) pointing out the administrative aspects of managing health concerns. Words like "problème" (problem), “rien” (nothing) and "vouloir" (want) may relate to challenges study participants face when trying to address health-related issues. **Topic 4** emphasizes financial and treatment challenges, with terms like "maladie" (illness), "traitement" (treatment), and "payer" (pay). Words like "peut-être" (perhaps) and "rare" may suggest uncertainties related to treatment access or affordability. **Topic 5** highlights personal and financial responsibility, with words like "prendre" (take), "devoir" (duty), and "salaire" (salary). The inclusion of "cout" (cost) and "aide" (help) may reflect the need for financial assistance in healthcare. "Maladie" (illness) and "médecin" (doctor) are present across several topics, underscoring that healthcare and illness management dominate the discussions. Also "assurance" (insurance) appears in several topics, suggesting that healthcare insurance is a pervasive topic for French-speaking study participants.

The **Italian** topic modeling output reflects recurring themes centered around financial burdens, healthcare insurance, and study participants’ experiences. Each topic sheds light on various aspects of managing illness, costs, and the healthcare system. **Topic 1** emphasizes the general challenges faced by study participants, with words like "problema" (problem), "riuscire" (succeed), and "cassa" (short for “cassa malattia”, health insurance) reflecting financial and personal struggles. The inclusion of "volere" (want) and "persona" (person) suggests a focus on individual desires and needs within the healthcare system. **Topic 2** focuses on healthcare and insurance, with terms like "assicurazione" (insurance), "medico" (doctor), and "invalidità" (disability) indicating the administrative and medical challenges that study participants face. Words like "pagare" (pay) and "complementare" (complementary) highlight financial concerns, particularly with medication and additional insurance costs. **Topic 3** centers around financial and insurance-related issues, with key terms like "cassa" (short for “cassa malattia”, health insurance), "malato" (patient), and "polizza" (policy) reflecting ongoing concerns about coverage and healthcare costs. "Invalidità" (disability) and "ricevere" (receive) suggest a focus on the benefits and support that study participants are trying to access. **Topic 4** highlights the intersection of work and healthcare, with "lavorare" (work), "pagare" (pay), and "assicurazione" (insurance) pointing to the financial pressures of balancing employment and medical expenses. The word "costo" (cost) underscores the financial burden, while "aiuto" (help) suggests the need for support in navigating these challenges. **Topic 5** focuses on personal experiences and financial struggles, with words like "pagare" (pay), "esperienza" (experience), and "assicurazione" (insurance) reflecting the financial difficulties study participants encounter. The inclusion of "marito" (husband) and "giuridico" (legal) suggests that study participants may also face familial and legal challenges when dealing with healthcare and insurance. Also in the Italian data, "assicurazione" and “cassa” (health insurance) appear across multiple topics, reflecting the significance of insurance in study participants' financial and healthcare experiences, and emphasizing the role of health insurance funds or social security.

The analysis of the **English** data reveals key topic focused on healthcare costs, work-related challenges, and the role of medical professionals in managing chronic illness. **Topic 1** could emphasize the psychological and physical demands of managing healthcare, with words like "think," "need," and "feel" indicating the emotional weight of navigating work and disease. **Topic 2** centers on decision-making and communication, with key terms like "tell," "need," and "doctor" suggesting conversations between study participants and healthcare providers. The presence of "time," "way," and "lot" reflects study participants' concerns about the allocation of time and resources. **Topic 3** focuses on professional healthcare interactions, with "doctor," "specialist," and "cost" underscoring the reliance on specialized care and the associated financial impact. Terms like "test," "month," and "disease" suggest a frequent need for medical testing. **Topic 4** highlights financial strain with the recurring terms "cost," "burden," and "work." Words like "year" and "disease" suggest long-term struggles with chronic illness management. **Topic 5** shifts the focus to overall well-being and lifestyle, with words like "start," "feel," "good," and "nutrition" pointing to the broader impacts of health on daily life. This topic suggests that managing health issues isn't only about finances but also involves a holistic approach to improving quality of life. "Cost" and "work" appear in multiple English topics, indicating that financial strain and employment are recurrent themes. Similarly, "doctor" and "disease" appear in several topics, possibly indicating the central role of healthcare professionals and chronic illness management in study participants’ narratives.

## **Narrative Examples**

### Money Issues

*"C’est pour ça que je suis obligé d’engager trois fois par semaine à temps complet / à journées complètes ça veut dire des journées de huit heures quelqu’un je paie 25 francs à l’heure, vous voyez à peu près les dépenses que ça occasionne et puis un jour par semaine elle est ici."* ("That’s why I have to hire someone three times a week, full-time—meaning eight-hour days. I pay 25 francs an hour, so you can imagine the kind of expenses that creates, and then one day a week she is here.") ***[Row 354, DEM 05].***

This passage illustrates the financial burden of chronic illness, where personal expenses go beyond covered care to ensure the management of daily life. It reflects the difficult personal decisions that must be made, which may lead to stress and frustration:

*"Quando hai una diagnosi così più nessuno ti assicura perché lo sanno già che per loro è un costo. […] è un costo per loro perché se io mi faccio le ricette per farmi massaggi […] per queste storpie di gambe che ho o cose così, la cassa malati deve pagare. […] per loro è una perdita, perciò non trovi nessuno che ti copre"* ("When you have such a diagnosis, no one will insure you anymore because they already know it's a cost for them. […] It’s a cost for them because if I write prescriptions for myself to get massages […] for these messed-up legs I have, or things like that, the health insurance company has to pay. I also pay the premium, but for them, it's a loss, so you can't find anyone who will cover you.") ***[Row 628, MSC 09].***

This passage highlights a perceived injustice in the Swiss health insurance system toward the chronically ill, who are left alone with the costs. This is driven by the insurers' economic interest in maximizing profits.

### Insurance – disability

One interviewee, describing her daily life after receiving disability status, explained how institutional support takes patience and time:

*"Oui, je reçois une rente AI pour ça. Et actuellement, je suis en train de faire des procedures, je ne sais pas si ça va fonctionner ou pas avec l'AI [Assurance Invalidité], pour voir si c’est possible d’avoir plus d’aide. Mais ça, ce sont des choses qui prennent du temps, et voilà, il faut être patient et espérer que l'AI reconnaisse tout ça, que je puisse recevoir des aides supplémentaires." (“Yes, I receive a disability pension for that. And currently, I'm going through some procedures, I don't know if it will work or not with the disability insurance, to see if it’s possible to get more assistance. But these things take time, and so, one has to be patient and hope that the disability insurance recognizes all this, so I can receive additional support.”)* ***[Row 1504, RD 29].***

This passage illustrates how navigating disability insurance systems can be a slow, uncertain, and emotionally taxing process. It underscores not only the administrative complexity involved, but also the precarity of relying on systems that may or may not recognize one’s condition as meriting additional support. The speaker’s hesitant, contingent phrasing captures a broader theme in the data: how bureaucratic opacity and delayed decisions compound financial stress with psychological strain, demanding patience without offering assurance.

Another quote shifts the perspective to a caregiving spouse, reflecting how the loss of work is distributed within family systems:

*"[…] Er hat ja noch Teilzeit, 40 %, gearbeitet, oder er arbeitet immer noch 40 %. Das heißt, an den Arbeitstagen konnte er selbstständig alles unternehmen […] Er hat mir erzählt, dass es einfach immer mehr Kraft braucht. […]" (“He still worked part-time, 40 percent, or he still does. That means on workdays he could manage everything independently […] but he told me it just takes more and more energy.”)* ***[Row 897, PDI 06].***

Here, the endurance of labor is reframed not as economic productivity but as accumulated fatigue. Part-time work becomes a strategy of survival, not autonomy.
